# Evaluation of mitochondria in oocytes following γ-irradiation

**DOI:** 10.1038/s41598-019-56423-w

**Published:** 2019-12-27

**Authors:** Qiaochu Wang, Jessica M. Stringer, Jun Liu, Karla J. Hutt

**Affiliations:** Biomedicine Discovery Institute, Department of Anatomy and Developmental Biology, Monash University, Melbourne, Australia

**Keywords:** Cell biology, Endocrinology

## Abstract

Standard cytotoxic cancer treatments, such as radiation, can damage and deplete the supply of oocytes stored within the ovary, which predisposes females to infertility and premature menopause later in life. The mechanisms by which radiation induces oocyte damage have not been completely elucidated. The objective of this study was to determine if γ-irradiation changes mitochondrial characteristics in oocytes, possibly contributing to a reduction in oocyte number and quality. Immature oocytes were collected from postnatal day (PN) 9–11 C57Bl6 mice 3, 6 and 24 hours after 0.1 Gy γ-irradiation to monitor acute mitochondrial changes. Oocytes were classified as small (>20 µm) or growing (40–60 µm). Mitochondrial membrane potential was lost in 20% and 44% of small oocytes (~20 µm) at 3 and 6 hours after γ-irradiation, respectively, consistent with the induction of apoptosis. However, mitochondrial mass, distribution and membrane potential in the surviving small oocytes were similar to the non-irradiated controls at both time points. At 24 hours after γ-irradiation, all mitochondrial parameters analysed within immature oocytes were similar to untreated controls. Mitochondrial parameters within growing oocytes were also similar to untreated controls. When mice were superovulated more than 3 weeks after γ-irradiation, there was a significant reduction in the number of mature oocytes harvested compared to controls (Control 18 ± 1 vs 0.1 Gy 4 ± 1, n = 6/16 mice, p < 0.05). There was a slight reduction in mitochondrial mass in mature oocytes after γ-irradiation, though mitochondrial localization, mtDNA copy number and ATP levels were similar between groups. In summary, this study shows that γ-irradiation of pre-pubertal mice is associated with loss of mitochondrial membrane potential in a significant proportion of small immature oocytes and a reduction in the number of mature oocytes harvested from adult mice. Furthermore, these results suggest that immature oocytes that survive γ-irradiation and develop through to ovulation contain mitochondria with normal characteristics. Whether the oocytes that survive radiation and eventually undergo meiosis can support fertility remains to be determined.

## Introduction

Conventional cytotoxic cancer treatments, including radiation and chemotherapy, can damage the oocytes and somatic cells within the ovary, leading to a reduction in the size of the ovarian follicular reserve, which predisposes females to infertility and premature menopause later in life^1^. With improving survival rates for many cancers, it is desirable to avoid these side-effects and devise new strategies to protect the long-term fertility and health of women post-treatment^2^. In order to achieve this goal, it is necessary to understand the mechanisms by which specific cancer treatments damage the ovary and induce oocyte death.

Studies have shown that ionising radiation induces DNA double strand breaks in the genomic DNA of oocytes, leading to their apoptosis^3,4^. Interestingly, evidence from somatic cells and cancer cell lines suggest that radiation may also damage mitochondria^5,6^. However, radiation-induced mitochondrial damage has not been evaluated in oocytes. It has also been proposed that lack of histones and limited capacity for DNA repair may make mitochondrial (mt) DNA more susceptible to radiotherapy than nuclear DNA^7,8^. Previous studies indicate that damage of mitochondria and mtDNA plays a key role in radiation toxicity in human fibroblasts^9^. Decreased ATP levels and deficiencies in the activity of the electron transport chain (ETC) complexes are commonly observed in different cell types after radiation, indicating mitochondrial damage^10,11^. Furthermore, an increase in mitochondrial mass and mtDNA content (copy number) has been observed after radiation treatment and is thought to be a compensatory mechanism to maintain normal mitochondrial function^12^.

Mitochondria are the powerhouse of the cell and comprise approximately 4–25% of total cell volume in eukaryotes and mature oocytes contain the largest number of mitochondria among different cell types^13,14^. Mitochondria play many important roles within the oocyte during maturation, fertilisation and preimplantation embryonic development, including synthesizing adenosine triphosphate (ATP), regulating redox and Ca2+ homeostasis and controlling apoptosis^15^. Because of this, alterations in mitochondrial number and function in oocytes may reduce oocyte quality and subsequently compromise embryonic development^16^. Furthermore, as mitochondria are maternally inherited, mtDNA damage, such as point mutations, may transferred to the next generation^17^. Therefore, it is important to examine the characteristics of mitochondria in oocytes following exposure to γ-irradiation. If cancer treatment-induced mitochondrial damage contributes to depletion of the ovarian reserve or loss of oocyte quality, then protection of mitochondria may represent a novel strategy for alleviating radiation and chemotherapy-mediated insult to the ovary^18^.

The objective of this study was to investigate the impact of γ-irradiation on mitochondria in immature oocytes. To do this, we used a mouse model in which prepubertal (PN9–11) female C57BL6 mice were untreated (controls) or exposed to whole body γ-irradiation at 0.1 Gy. At 3, 6 and 24 hours after treatment, immature oocytes were collected to monitor acute changes in mitochondrial mass and distribution, function (ATP levels) and mtDNA copy number. In addition, γ-irradiated mice were held for a minimum of 3 weeks to determine if damage inflicted on immature oocytes persists in mature oocytes.

## Results

### Mitochondrial distribution and mass were not affected by γ-irradiation in immature oocytes

Immature oocytes were collected from mice 3, 6 and 24 hours following γ-irradiation (or from untreated controls) and classified according to the size. Oocytes with a diameter ~20 μm (range 12–21 μm) were defined as small oocytes from primordial or primary follicles (Supplemental Fig. 1A,B). Oocytes with a diameter ~50 μm (range 39–62 μm) were defined as growing oocytes from secondary follicles (Supplemental Fig. 1).

**Figure 1:**
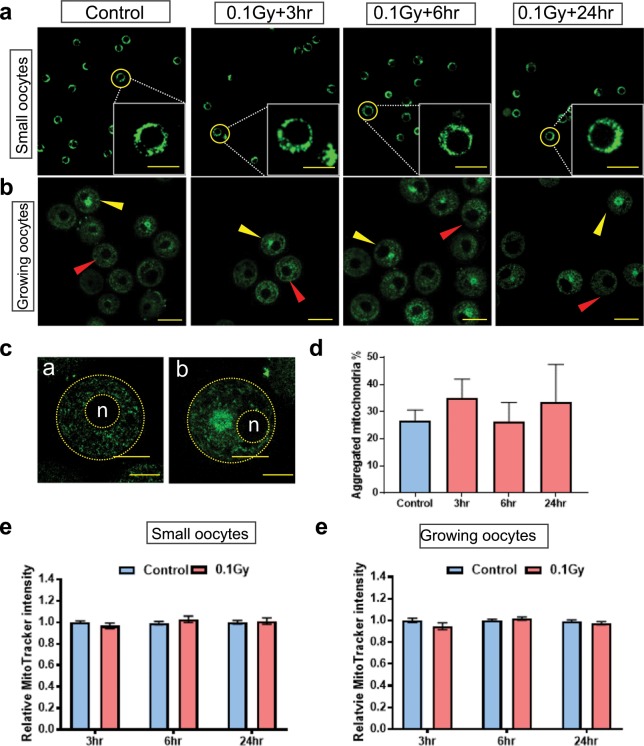
The distribution of mitochondria in small and growing immature oocytes from untreated and γ-irradiated mice. Immature oocytes were isolated from untreated control mice, or 3, 6 and 24 hours after γ-irradiation and mitochondrial distribution was assessed using MitoTracker Green (green) (n = 3–4 mice per group). (**a**) Mitochondria were distributed throughout the cytoplasm in small oocytes. Scale bar = 20 μm. (**b**) Mitochondria were either evenly distributed in the cytoplasm (red arrow head) or aggregated close to nucleus (yellow arrow head) in growing oocytes. Scale bar = 50 μm. (**c**) Enlarged view of homogenous mitochondrial distribution (**a**) and aggregated distribution (**b**). n = nucleus. Scale bar = 20 μm. (**d**) Percentage of growing oocytes with aggregated mitochondria for controls (n = 158) and 3 (n = 61), 6 (n = 54), 24 (n = 36) hours after γ-irradiation. No significant differences were observed (Kruskal-Wallis test, p-value > 0.05). (**e**) Relative MitoTracker intensity in small oocytes at 3 (n = 41/32), 6 (n = 59/21) and 24 (n = 39/23) hours after γ-irradiation compared to control. No significant differences were observed between control and γ-irradiated groups (*t-*test, p-value > 0.05). (**f**) Relative MitoTracker intensity in growing oocytes, at 3 (n = 48/42), 6 (n = 59/64) and 24 (n = 61/63) hours after γ-irradiation compared to control. No significant differences were observed between control and γ-irradiated groups (*t-*test, p-value > 0.05).

To assess mitochondrial distribution, live immature oocytes were incubated with the fluorescent dye MitoTracker Green. In small oocytes, mitochondria were distributed throughout the cytoplasm and no differences were observed in the localisation pattern between oocytes from untreated and γ-irradiated mice at any time point (Fig. 1a). In growing oocytes, mitochondria were distributed in two distinct patterns; either evenly distributed in the cytoplasm or aggregated close to nucleus (Fig. 1b,c). The homogenous and aggregated distribution patterns appeared in both control and γ-irradiated oocytes and no differences in the percentage of oocytes with aggregated mitochondria were observed between groups at any time point (Fig. 1d).

To assess whether mitochondrial abundance was affected by γ-irradiation, relative MitoTracker Green fluorescence intensity was quantified. There was no significant difference γ-irradiated groups relative to the control for small and growing oocytes at 3, 6 and 24 hours (Fig. 1e,f).

### Mitochondrial membrane potential was transiently impaired in immature oocytes following γ-irradiation

**Figure 2:**
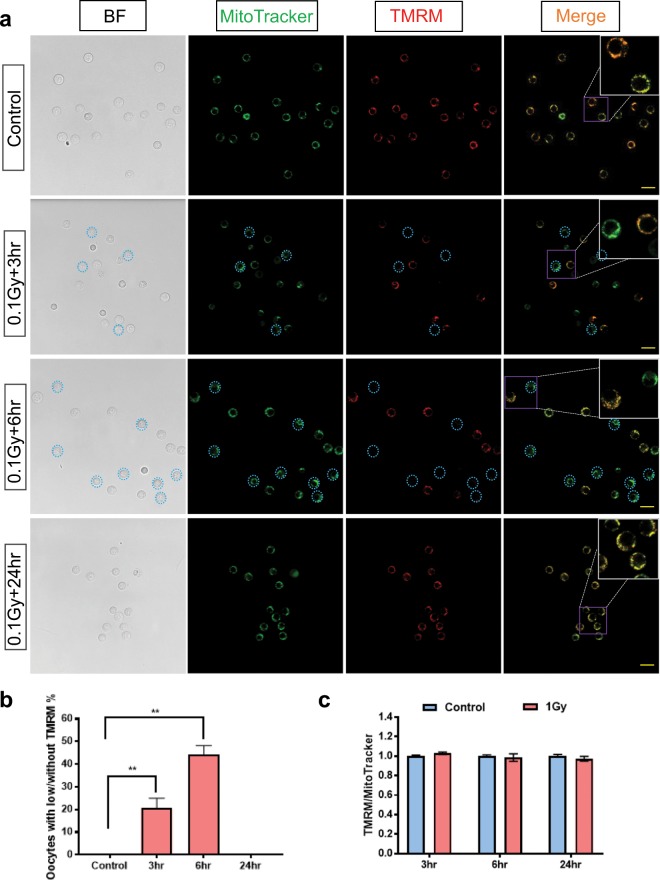
Membrane potential of mitochondria in small immature oocytes from untreated and γ-irradiated mice. Small oocytes were isolated from untreated control mice, or 3, 6 and 24 hours after γ-irradiation and mitochondrial membrane potential was assessed using TMRM. (**a**) Representative confocal images of small oocytes from untreated mice and γ-irradiated mice at 3, 6 and 24 hours after treatment. Scale bar = 40 μm. (**b**) Percentage of small oocytes with low or without TMRM in untreated oocytes (n = 124) and γ-irradiated oocytes at 3 (n = 32), 6 (n = 39) and 24 (n = 24) hours after γ-irradiation. Ordinary one-way ANOVA, Dunnett’s multiple comparisons test, **p-value < 0.01. (**c**) Relative TMRM/MitoTracker ratio in small oocytes with TMRM signal at 3 (n = 41/26), 6 (n = 59/21) and 24 (n = 39/23) hours after irradiation. No significant differences were observed between control and γ-irradiated groups (*t-*test, p-value > 0.05).

**Figure 3:**
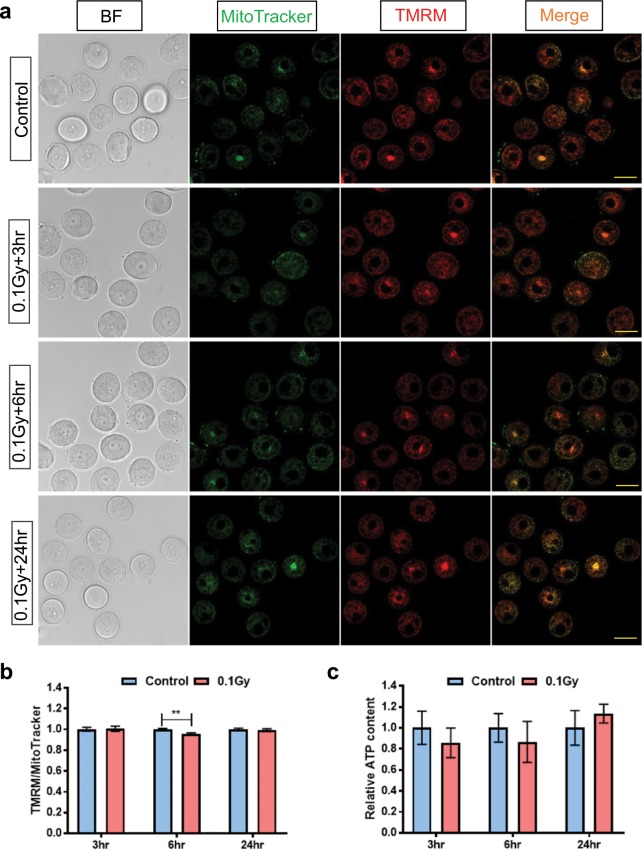
Membrane potential of mitochondria in growing immature oocytes from untreated and γ-irradiated mice. Growing oocytes were isolated from untreated control mice, or 3, 6 and 24 hours after γ-irradiation and mitochondrial membrane potential was assessed using TMRM. (**a**) Representative confocal images of growing oocytes from untreated mice and γ-irradiated mice at 3, 6 and 24 hours after treatment. Scale bar = 50 μm. (**b**) Relative TMRM/MitoTracker ratio in growing oocytes at 3 (n = 48/42), 6 (n = 59/64) and 24 (n = 61/63) hours after irradiation. Experiments were performed in triplicate. A significant decrease of TMRM intensity was observed only at 6 hours after γ-irradiation. Mann-Whitney test, **p-value < 0.01. No significant differences were observed between control and γ-irradiated groups at 3 and 24 hours (Mann-Whitney test and *t-*test, respectively, p-value > 0.05). (**c**) Relative ATP content in growing oocytes 3 (n = 7/7), 6 (n = 7/8) and 24 (n = 4/4) hours after γ-irradiation. No significant differences were observed between control and γ-irradiated groups (*t-*test, p-value > 0.05).

We next determined whether mitochondrial membrane potential (ΔΨm) was altered in immature oocytes from γ-irradiated mice by staining with TMRM, a cell-permeant dye that accumulates in active mitochondria with intact membrane potential (Figs. 2a and 3a). Oocytes were simultaneously stained with MitoTracker Green to confirm to presence of mitochondria and normalise TMRM levels to mitochondrial abundance.

Whilst TMRM staining was detectable in all control small oocytes, at 3 and 6 hours after γ-irradiation, mitochondrial membrane potential was lost in 20% and 44% of small oocytes, respectively (Fig. 2b). Loss of mitochondrial membrane potential is consistent with apoptosis^19^. In contrast, at the later time point of 24 hours after γ-irradiation, all oocytes analysed retained mitochondrial membrane potential (Fig. 2b) as indicated by detectable TMRM staining. We then quantified TMRM intensity in those small oocytes with detectable TMRM staining. No significant differences were observed between control and γ-irradiated groups at each time point (p > 0.05) (Fig. 2c).

TMRM staining was observed in all growing oocytes in both control and γ-irradiated groups (Fig. 3a). A slight decrease in TMRM intensity relative to controls was observed at 6 hours after γ-irradiation (Fig. 3b). We also evaluated ATP levels in growing oocytes. No differences were observed in the relative ATP content in control and γ-irradiated groups at any time point (Fig. 3c).

### Mitochondrial characteristics were similar in mature oocytes ovulated by control mice or mice γ-irradiated 3 weeks earlier

**Figure 4:**
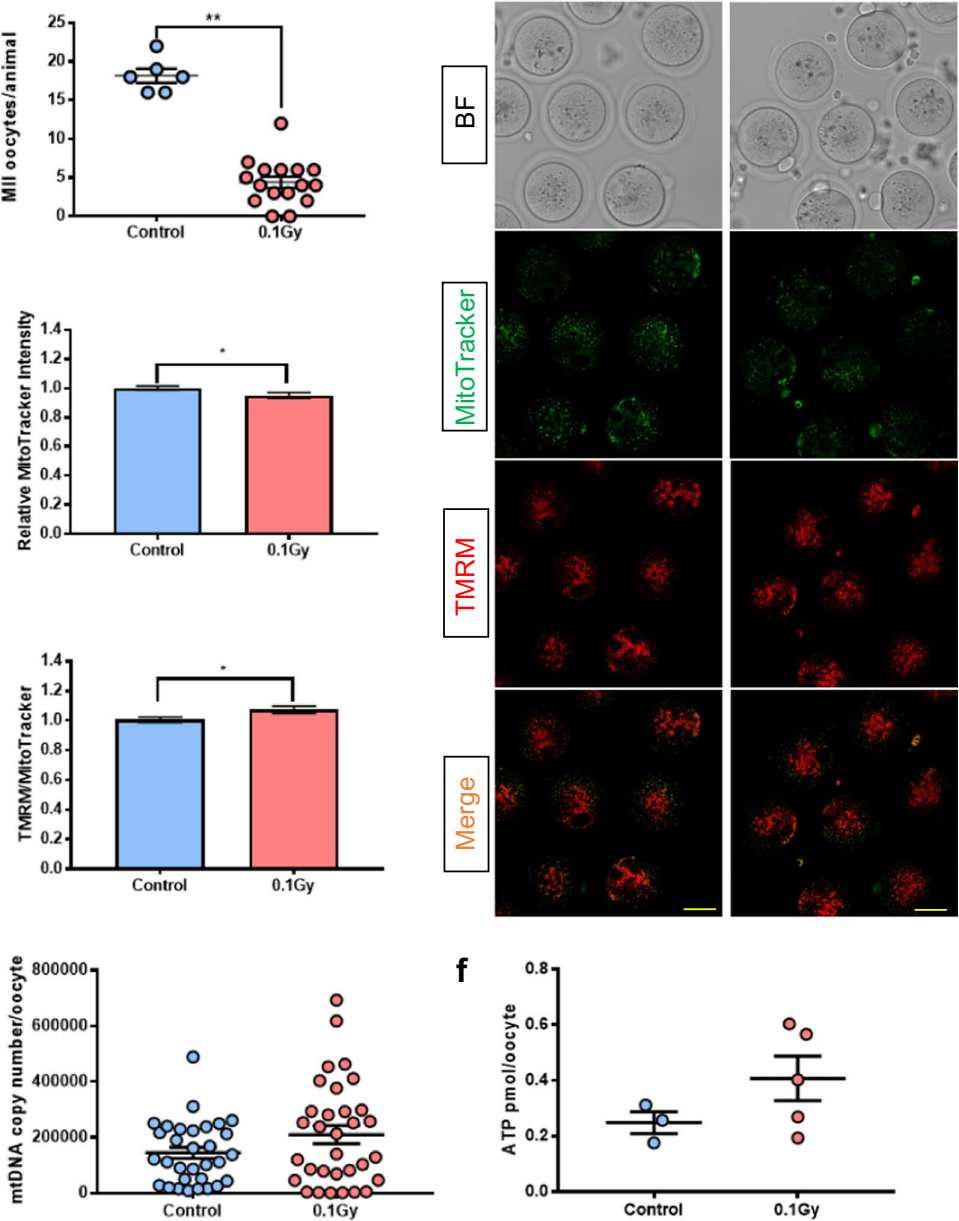
Mitochondria in mature oocytes. Mice were untreated (controls) or γ-irradiated at PN9-11 and then allowed to develop to sexual maturity before being super ovulated. mtDNA copy number was assessed in individual mature oocytes. (**a**) Number of oocytes harvested from adult mice. Each dot represents one animal, animal number n = 6/16 for control and γ-irradiated groups respectively. *t-*test, **p-value < 0.01. (**b**) Representative confocal images of mature oocytes. Scale bar = 50 μm. (**c**) Relative MitoTracker intensity of mature oocytes from untreated control mice (n = 44 oocytes) and γ-irradiated mice (n = 36 oocytes). *t-*test, p-value > 0.05. (**d**) TMRM to MitoTracker ratio in mature oocytes, oocytes number n = 44 in control group and n = 36 in irradiated group. *t-*test, *p-value < 0.05. (**e**) mtDNA copy number in mature oocytes in control (n = 31 oocytes) and γ-irradiated (n = 32 oocytes) groups. No significant differences were observed, Mann-Whitney test, p-value > 0.05. (**f**) ATP content in mature oocytes. Oocytes were pooled into groups of 10 ATP measurement. Oocytes were collected from three and five biological replicates for control and γ-irradiated groups respectively. No significant differences were observed, *t*-test, *p-value < 0.05.

To determine if γ-irradiation of immature oocytes generated changes in mitochondria that persist in mature oocytes, mice were super ovulated more than 3 weeks after γ-irradiation and MII oocytes were collected. Notably, the number of mature oocytes harvested from γ-irradiated mice was dramatically reduced compared to controls (Control 18 ± 1 vs 0.1 Gy 4 ± 1, n = 6/16 mice, p < 0.01), with 2 mice failing to ovulate (Fig. 4a). Staining with MitoTracker Green revealed a relatively homogeneous distribution pattern of mitochondria in the ovulated oocytes from control and γ-irradiated mice. A slight decrease in MitoTracker Green intensity relative to controls was observed in the γ-irradiated group (Fig. 4b,c). This likely resulted in the slight increase in TMRM staining intensity relative to MitoTracker Green that was observed in ovulated oocytes from γ-irradiated mice compared to controls (Fig. 4b,d).

Notably, we found that mtDNA copy number was similar in MII oocytes from control and γ-irradiated mice. Similarly, ATP level in γ-irradiated mice was not significantly different from controls (Fig. 4f).

## Discussion

The goal of this study was to evaluate the impact of γ-irradiation on the mitochondria in oocytes. Mitochondrial distribution is thought to be important for meeting the energy requirements of cellular activities^20^, therefore we used MitoTracker Green to localise mitochondria in small and growing oocytes from control and irradiated mice. We found that mitochondria were distributed evenly throughout the cytoplasm of small oocytes, consistent with a previous report^21^, and we did not observe any change of mitochondrial localisation following γ-irradiation. In growing oocytes, mitochondria were found to be distributed in either a homogeneous or an aggregated pattern, though the functional significance of the two patterns is not clear. Importantly, the proportion of oocytes with each localisation pattern in growing oocytes from control and γ-irradiated mice was similar.

Mitochondria are highly dynamic organelles in eukaryotes, continually undergoing fusion and fission^22^. The balance of these two processes maintains the overall population of mitochondria and is related to mtDNA stability, energy production, apoptosis^22^. It is proposed that an increase in mitochondrial abundance (number) is a mechanism of compensation for oxidative defects and is commonly observed in somatic cells following exposure to γ-irradiation^5,23^. In contrast, we did not detect any changes in mitochondrial abundance as indicated by MitoTracker fluorescent intensity in small oocytes at 3, 6 or 24 hours after γ-irradiation. However, it is possible that the timeframe we picked in our study was too late, as one previous study of mouse oocytes found that mitochondrial number dropped 2 minutes after whole body x-irradiation at 200r and was restored in the next few minutes^24^.

Mitochondrial membrane potential generated by proton pumping is essential for ATP synthesis and storage^25^. Thus, a sharp and persistent change of mitochondrial membrane potential may reduce cellular viability^19,26^. In this study, we found that the TMRM fluorescent signal emitted by cells was lost in 20% and 44% of small oocytes at 3 and 6 hours after γ-irradiation, respectively. This finding indicates that the mitochondria in a cohort of oocytes were compromised by γ-irradiation and these oocytes were likely undergoing apoptosis^19^. Additional studies are required to determine if this is due to direct damage of the mitochondria by γ-irradiation or by the induction of apoptosis (e.g. as a result of damage to nuclear DNA). Interestingly, all small oocytes had detectable TMRM staining at 24 hours after γ-irradiation, suggesting the surviving oocytes can repair damage sustained by the mitochondria, or that the mitochondria of the surviving oocytes were in fact not damaged by the 0.1 Gy dose of γ-irradiation used in this study. Quantification of the relative TMRM intensity in the small oocytes showed no differences at any time point, suggesting the latter possibility may be true.

In contrast to small oocytes, γ-irradiation did not appear to be associated with lethal loss of mitochondrial membrane potential in growing oocytes, highlighting well-documented intrinsic differences in the radio-sensitivities of these two populations^20^. The ratio of TMRM to MitoTracker showed a very slight decrease at 6 hours after γ-irradiation in the growing oocyte population, but the subtlety of the change makes it unclear if this would have a meaningful biological consequence. Indeed, ATP levels were maintained in growing oocytes after γ-irradiation, indicating that mitochondria were functionally similar to non-irradiated controls.

To determine if damage inflicted on immature oocytes persists in mature oocytes, γ-irradiated mice were held for a minimum of 3 weeks (the period that required for immature oocytes to fully develop) before being superovulated. We found the number of mature oocytes from γ-irradiated mice was significantly lower than controls. This observation is in keeping with previous work demonstrating a reduction in the size of the ovarian reserve following γ-irradiation due to oocyte apoptosis^3^. It is also possible that γ-irradiation reduces the ability of mice to respond to exogenous hormonal stimulation, perhaps by damaging the hypothalamic–pituitary–gonadal axis, though the low dose of γ-irradiation used in this study make this hypothesis unlikely. We did observe a slight decrease in MitoTracker staining intensity, and concomitant increase in TMRM staining intensity, in γ-irradiated ovulated oocytes relative to controls, but whether such a small relative change is biologically or functionally meaningful is unclear. Furthermore, mitochondrial localisation, mtDNA copy number and ATP levels in these oocytes were similar to oocytes from non-irradiated mice. Overall, the results suggest that mitochondria function was not impaired in mature oocytes from γ-irradiated mice.

There were some limitations associated with this study. For example, this model used a very low dose of γ-irradiation. This was necessary to ensure that a cohort of oocytes survived to allow evaluation. Notably, girls and women would receive much higher doses and we cannot rule out the possibility that higher doses would lead to persistent effects on the mitochondria in oocytes. Furthermore, whilst we determined that mitochondria were capable of generating levels of ATP in γ-irradiated similar to controls, we did not evaluate ROS levels or other impacts, such as calcium signalling. Additionally, damage to mtDNA was not directly assessed. Thus, further work is required to determine if mitochondria are fully functional and undamaged.

In conclusion, our study demonstrated immature oocytes that survived γ-irradiation and developed through to ovulation contained apparently healthy mitochondria, at least to the extent of generating normal levels of ATP. Future studies should focus on establishing whether these oocytes can support normal healthy pregnancy. These promising findings may provide guidance to preserve female fertility when making therapeutic regimen for female cancer patients.

## Materials and Methods

### Animals, treatments and oocyte collection

C57BL/6J mice were housed in a high-barrier facility (Monash University ARL) with controlled temperature and 12 h light: 12 h dark cycle. All mice had free access to water and food. All animal procedures and experiments were performed in accordance with the NHMRC Australian Code of Practice for the Care and Use of Animals and approved by the Monash Animal Research Platform Animal Ethics Committee. Prepubertal female mice (PN9-11, n = 3–4 mice/control and time point post γ-irradiation), an age at which ovaries contain an abundance of small oocytes, were exposed to whole body γ-irradiation at 0.1 Gy. This dose of γ-irradiation has been shown to induce nuclear DNA damage in oocytes, but approximately 50% survive^27^. To collect immature oocytes, ovaries were harvested 3, 6, 24 hours after γ-irradiation and digested in 0.25% trypsin (SM-203-C, Merck) for 13 minutes and then 200 μl 10% FBS (12003 C, Sigma-Aldrich) in M2 (M7167; Sigma-Aldrich) was added to cease digestion. Oocytes were measured using Las x software (Leica). Oocytes with a diameter ~20 μm were defined as small oocytes from primordial or primary follicles. Oocytes with a diameter ~50 μm were defined as growing oocytes from secondary follicles. To collect mature oocytes, untreated control and γ-irradiated mice were held for a minimum of 3 weeks (the period that required for immature oocytes to fully develop) and then treated with an intraperitoneal injection of with pregnant mare serum gonadotrophin (5IU PMSG; Intervet) followed 44–48 hours later by human chorionic gonadotropin (5 IU hCG; Intervet). After 12–16 hours, cumulus-oocyte complexes were collected from oviducts and mature oocytes (MII stage) were denuded by digestion in M2 media containing 0.3% hyaluronidase (Sigma-Aldrich).

### Assessment of mitochondrial membrane potential

Mitochondrial membrane potential was measured in single live oocytes using the low toxicity fluorescent dye tetramethyl rhodamine methyl ester perchlorate (TMRM, T668, ThermoFisher)^28^. TMRM is a cell-permeant dye that accumulates in active mitochondria with intact membrane potential. Briefly, denuded oocytes were incubated with 25 nM TMRM diluted in M2 medium at 37 °C for 30 minutes, washed 3 times in fresh M2 medium, mounted on the dish with glass bottom and then observed under a laser-scanning confocal microscope (SP8, Leica). TMRM was excited using the 552 nm laser line and fluorescence measured using a 563–627 nm band pass filter. A region encompassing the entire oocyte diameter was used to provide an average intensity of fluorescence within the oocyte. An identical region was used for each oocyte in all experiments. The plane of focus in which the oocyte diameter was largest was assumed to be the centre and was selected for image capture and analysis. The acquired images were processed and analysed using the ImageJ (NIH) open source software. For each image, the fluorescence intensity per single cell was calculated and expressed as the ratio of actual intensity to the mean intensity of control group. Experiments were repeated a minimum of three separate times (representing biological replicates) and data are expressed as mean +/− SEM. The number of oocytes analyzed is stated in the appropriate figure legend.

### Assessment of mitochondrial distribution and mass

Mitochondrial distribution and mass were evaluated using MitoTracker Green (M7514, ThermoFisher), which stains mitochondria regardless of mitochondrial membrane potential. Briefly, denuded oocytes from unprimed or gonadotropin-stimulated mice were incubated with 200 nM MitoTracker diluted in M2 at 37 °C for 30 minutes, washed 3 times in fresh M2, mounted on the dish with glass bottom and then observed under a laser-scanning confocal microscope (SP8, Leica). Mitotracker Green was excited using the 488 nm laser line and fluorescence measured using a 495–523 nm bandpass filter. The acquired images were processed and analysed using the ImageJ (NIH) open source software as described above. For each image, the fluorescence intensity per single oocyte was calculated and expressed as the ratio of actual intensity to the mean intensity of control group. Experiments were repeated a minimum of three separate times and data are expressed as mean +/− SEM for experiment.

### ATP quantification

Denuded oocytes (10 pooled/group) collected from unprimed or gonadotropin-stimulated mice were collected in 50 μl filtered ultrapure water and stored at −80 °C until use. To prepare standards, 10^−7^ M ATP standard stock was obtained from ENLITEN® ATP Assay System Bioluminescence Detection Kit (Promega) and was diluted with filtered ultrapure water. ATP levels were assayed using the Adenosine 5¢-triphosphate (ATP) bioluminescent somatic cell assay kit (FLASC, Sigma) according to the manufacturer’s instructions. Briefly, 100 μl ATP Assay Mix Working Solution was added to a 96-well plate (reaction vial) (M0187, Greiner) and allowed to stand at room temperature for 3 minutes. Somatic Cell ATP Releasing Reagent (100 μl), 50 μl filtered ultrapure water and 50 μl sample were added to a new tube and 100 μl was transferred to the reaction vial. ATP concentration was measured immediately using a luminometer (BMG, Clariostar, 76G58). Data are expressed as ATP content relative to the mean of controls or pmol/oocyte. Experiments were repeated a minimum of three separate times.

### Quantification of mtDNA copy number

mtDNA copy number was assayed on single oocytes as previously described^29^ with some modifications. Briefly, total DNA of single oocytes were isolated using 10 μl lysis buffer containing 50 mM tris-HCl (pH 8.5), 0.1 mM EDTA, 0.5% Tween-20 and 200 μg/ml proteinase K. Samples were incubated at 55 °C for 2 hours and then 95 °C for 10 minutes to inactivate the proteinase K. Six serial dilutions of stock plasmid (a.pngt from Rebecca Robker, The Robinson Research Institute, School of Medicine, The University of Adelaide, Australia) were used to generate the standard curve. Real-time quantitative PCR was performed in triplicate using 5′-CGTTAGGTCAAGGTGTAGCC-3′ and 5′-CCAAGCACACTTTCCAGTATG-3′ primers and SYBR green PCR master mix (Qiagen) and a 384-well real-time PCR machine (CFX384, Bio-Rad). The reaction conditions were 95 °C for 2 minutes and then 40 cycles of 95 °C for 5 seconds and 60 °C for 10 seconds. The number of oocytes analyzed is stated in the appropriate figure legend.

### Statistical analysis

Data were analysed using with GraphPad Prism Software. For comparison of two groups, normally distributed data were analysed by *t-*test, whilst non-normally distributed data were analysed by Mann-Whitney test. More than two groups of normally distributed data were analysed by ordinary one-way ANOVA followed by Dunnett’s multiple comparisons test, whilst non-normally distributed data were analysed by Kruskal-Wallis test. All data were presented as mean ± SEM. Statistical significance was denoted by *p-value < 0.05, **p-value < 0.01.

## Supplementary information

Supplemental Figure 1

## Data Availability

Materials, data and associated protocols are available on request.
