# Supplementary material for: Evaluation of mitochondria in oocytes following γ-irradiation
Source: Sci Rep. 2019 Dec 27;9:19941. doi: 10.1038/s41598-019-56423-w (PMC6934861; doi:10.1038/s41598-019-56423-w)
Supplement: Supplementary file 1 — Supplemental Figure 1 [file 41598_2019_56423_MOESM1_ESM.pdf]

Title: Evaluation of mitochondria in oocytes following γ-irradiation

Authors: Qiaochu Wang[1], Jessica M. Stringer[1], Jun Liu[1] and Karla J. Hutt1*.

1. Biomedicine Discovery Institute, Department of Anatomy and Developmental Biology, Monash University, Melbourne, Australia.

* Correspondence to karla.hutt@monash.eu

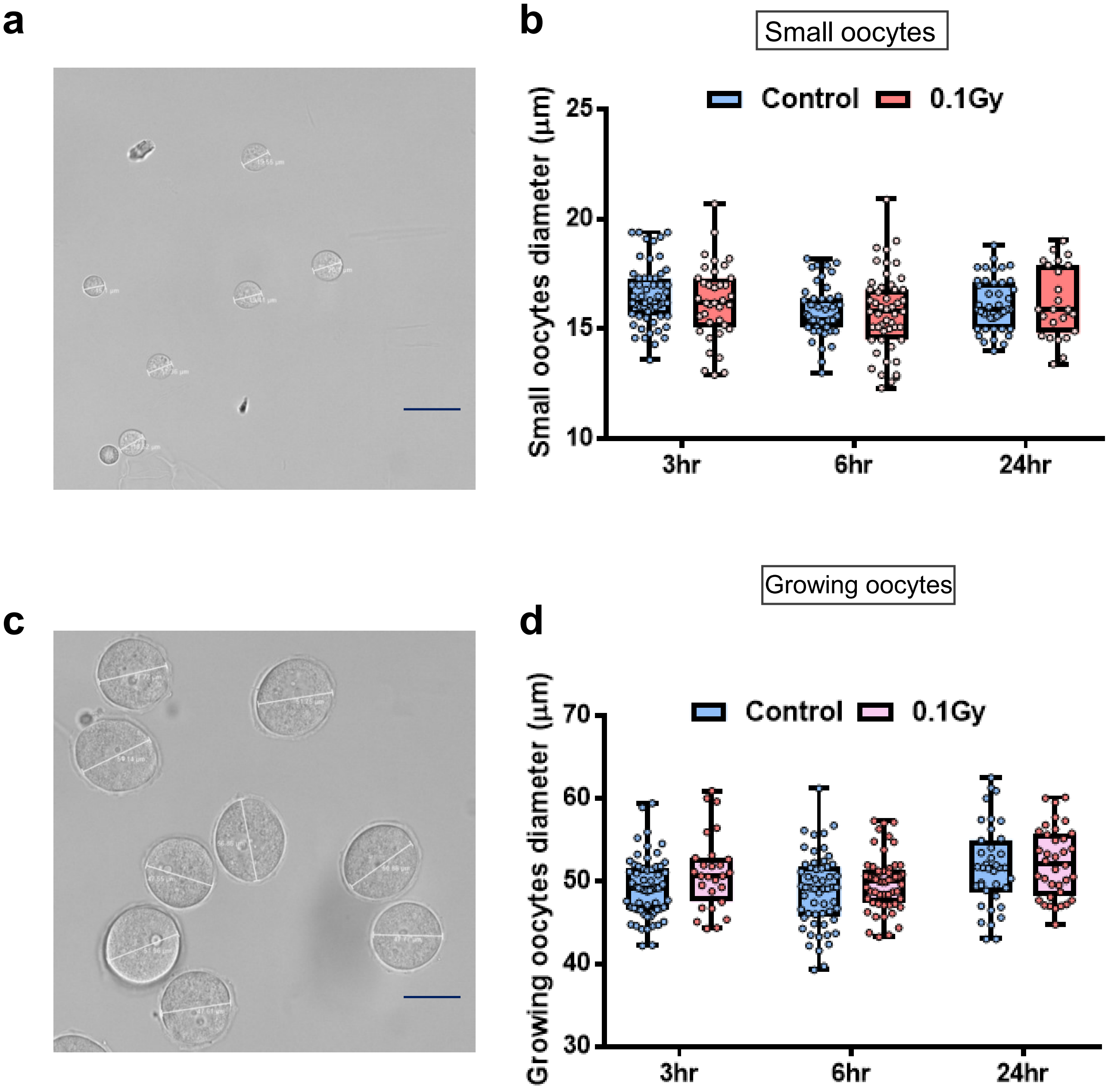

Supplemental Figure 1. Classification of oocytes according to size. A, Small oocytes (diameter ~20µm) were isolated from primordial or primary follicles. Scale bar = 50µm. B, Diameter of individual small oocytes, used for immunofluorescence analysis. Oocyte number at 3 (n=55/37), 6 (n=48/50) and 24 hours (n=42/25) for control and 0.1Gy γ-irradiated, respectively. C, Growing oocytes were isolated from secondary follicles. Scale bar = 50µm. D, Diameter of individual growing oocytes, used for immunofluorescence analysis. Oocyte number at 3 (n=64/61), 6 (n=56/54) and 24 (n=38/36) hours for control and γ-irradiated, respectively. Oocytes were collected from a minimum of 3 separate mice for each group.
